# Supplementary material for: Unsupervised correction of gene-independent cell responses to CRISPR-Cas9 targeting
Source: BMC Genomics. 2018 Aug 13;19:604. doi: 10.1186/s12864-018-4989-y (PMC6088408; doi:10.1186/s12864-018-4989-y)
Supplement: Supplementary file 1 — Table S1. Project Score cell lines included in the study with annotations and screening description. Table S2. Quantification of copy number-associated bias before and after CRISPRcleanR correction. Table S3. Recall reduction following CRISPRcleanR correction across control gene-sets and cell lines. Table S4. Recall reduction post CRISPRcleanR correction across controls (mean-variance modeling). Table S5. Cancer driver gene dependencies following CRISPRcleanR correction. Table S6. List of gene signatures downloaded from MSigDB and used as positive controls. (ZIP 6330 kb) [file 12864_2018_4989_MOESM1_ESM.zip › Supp.Figure S5.pdf]

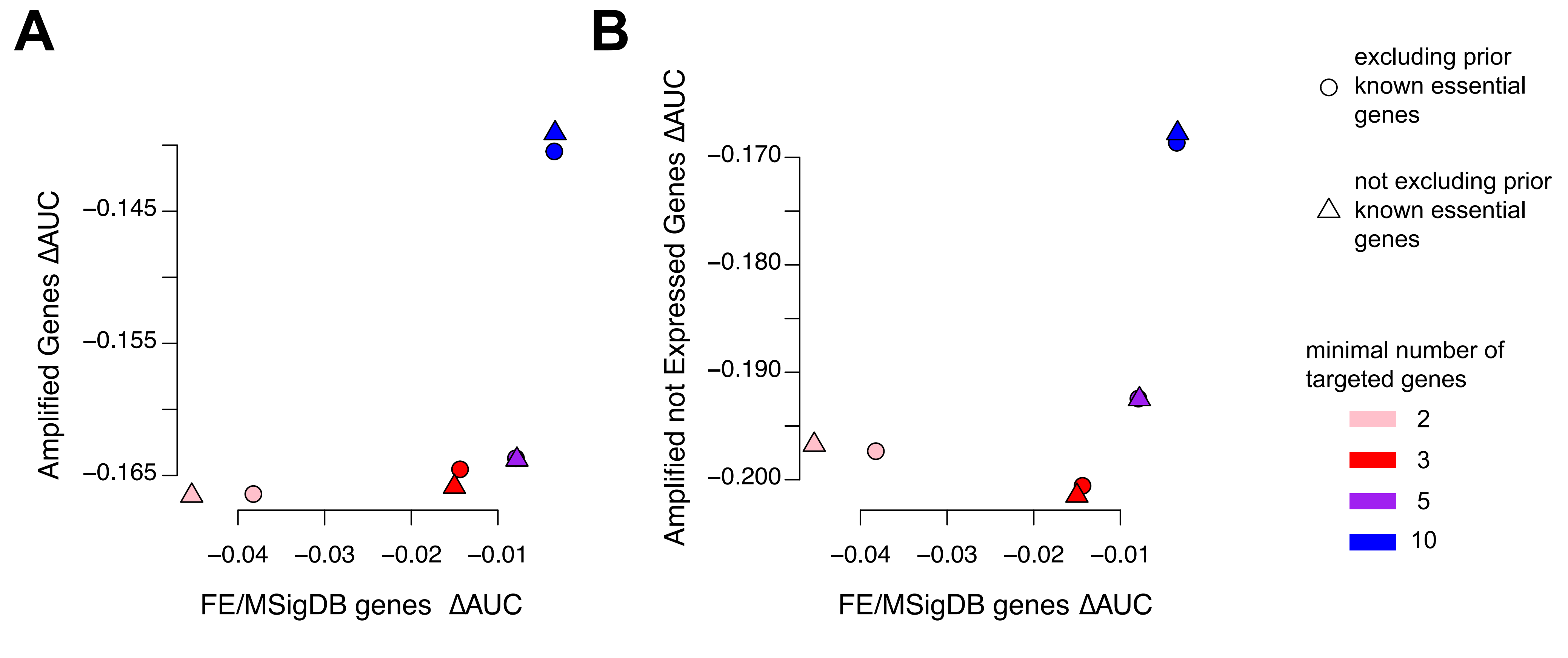
A
B
Amplified Genes ΔAUC
−0.145
−0.155
−0.165
−0.04
−0.03
−0.02
−0.01
FE/MSigDB genes ΔAUC
Amplified not Expressed Genes ΔAUC
−0.170
−0.180
−0.190
−0.200
−0.04
−0.03
−0.02
−0.01
FE/MSigDB genes ΔAUC
excluding prior known essential genes
not excluding prior known essential genes
minimal number of targeted genes
2
3
5
10
