# Supplementary material for: Unsupervised correction of gene-independent cell responses to CRISPR-Cas9 targeting
Source: BMC Genomics. 2018 Aug 13;19:604. doi: 10.1186/s12864-018-4989-y (PMC6088408; doi:10.1186/s12864-018-4989-y)
Supplement: Supplementary file 1 — Table S1. Project Score cell lines included in the study with annotations and screening description. Table S2. Quantification of copy number-associated bias before and after CRISPRcleanR correction. Table S3. Recall reduction following CRISPRcleanR correction across control gene-sets and cell lines. Table S4. Recall reduction post CRISPRcleanR correction across controls (mean-variance modeling). Table S5. Cancer driver gene dependencies following CRISPRcleanR correction. Table S6. List of gene signatures downloaded from MSigDB and used as positive controls. (ZIP 6330 kb) [file 12864_2018_4989_MOESM1_ESM.zip › Supp.Figure S8.pdf]

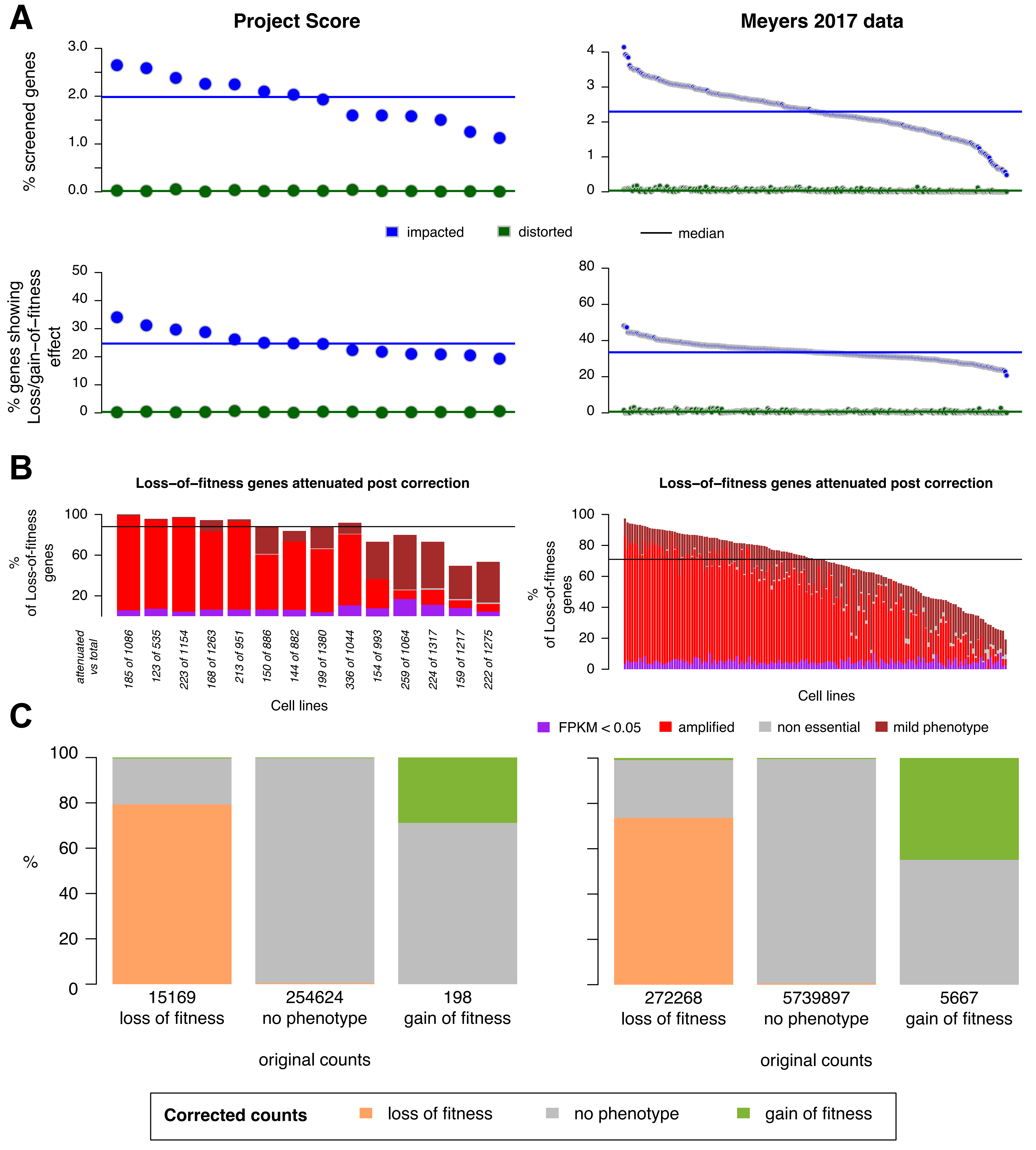

A
Project Score
Meyers 2017 data
% screened genes
impacted
distorted
median
% genes showing Loss/gain-of-fitness effect
B
Loss-of-fitness genes attenuated post correction
% of Loss-of-fitness genes
attenuated vs total
185 of 1086
123 of 535
223 of 1154
168 of 1263
213 of 951
150 of 886
144 of 882
199 of 1380
336 of 1044
154 of 993
259 of 1064
224 of 1317
159 of 1217
222 of 1275
Cell lines
C
FPKM < 0.05
amplified
non essential
mild phenotype
%
15169
loss of fitness
254624
no phenotype
198
gain of fitness
272268
loss of fitness
5739897
no phenotype
5667
gain of fitness
original counts
Corrected counts
loss of fitness
no phenotype
gain of fitness
