# Supplementary figures and images for: Unsupervised correction of gene-independent cell responses to CRISPR-Cas9 targeting
Source: BMC Genomics. 2018 Aug 13;19:604. doi: 10.1186/s12864-018-4989-y (PMC6088408; doi:10.1186/s12864-018-4989-y)

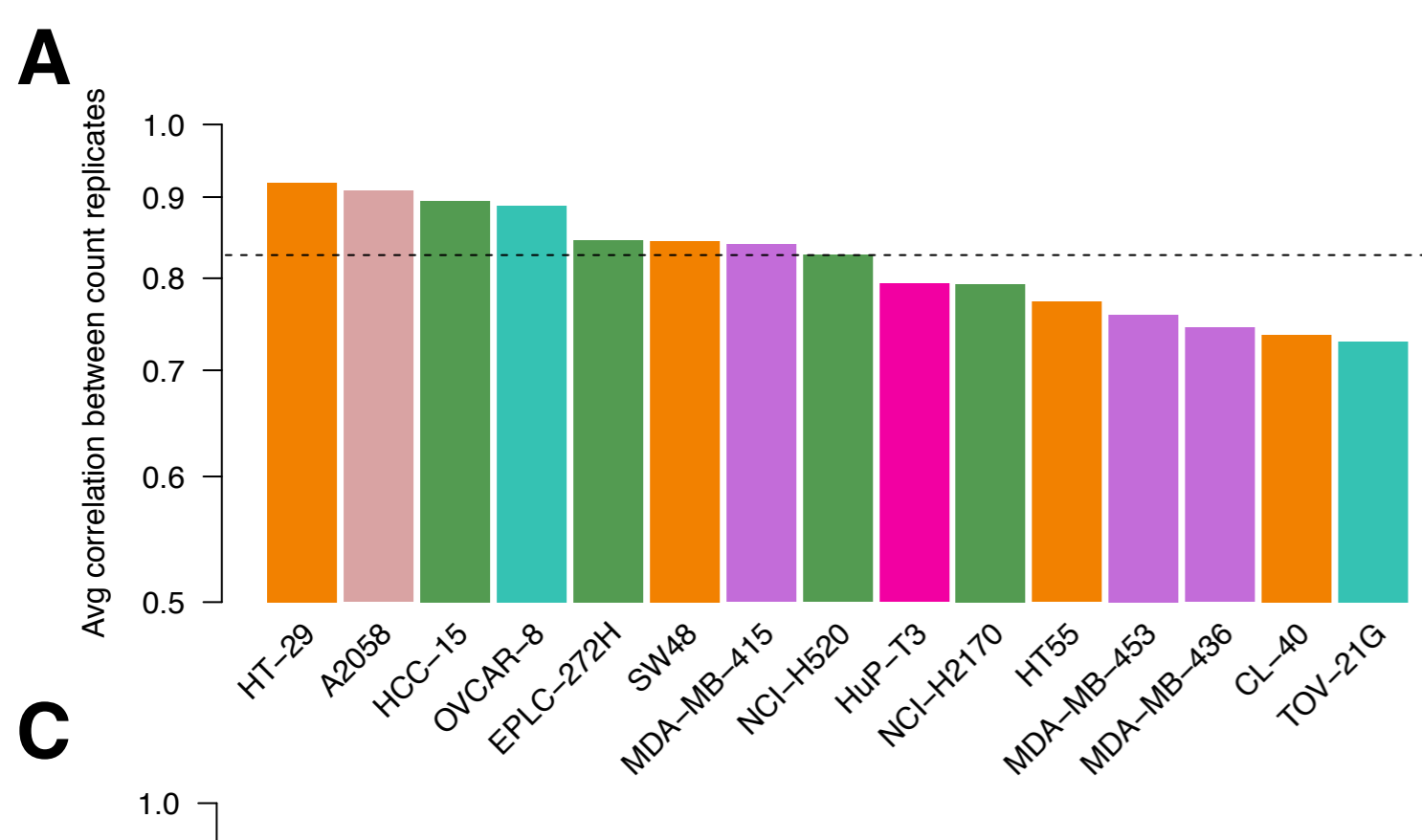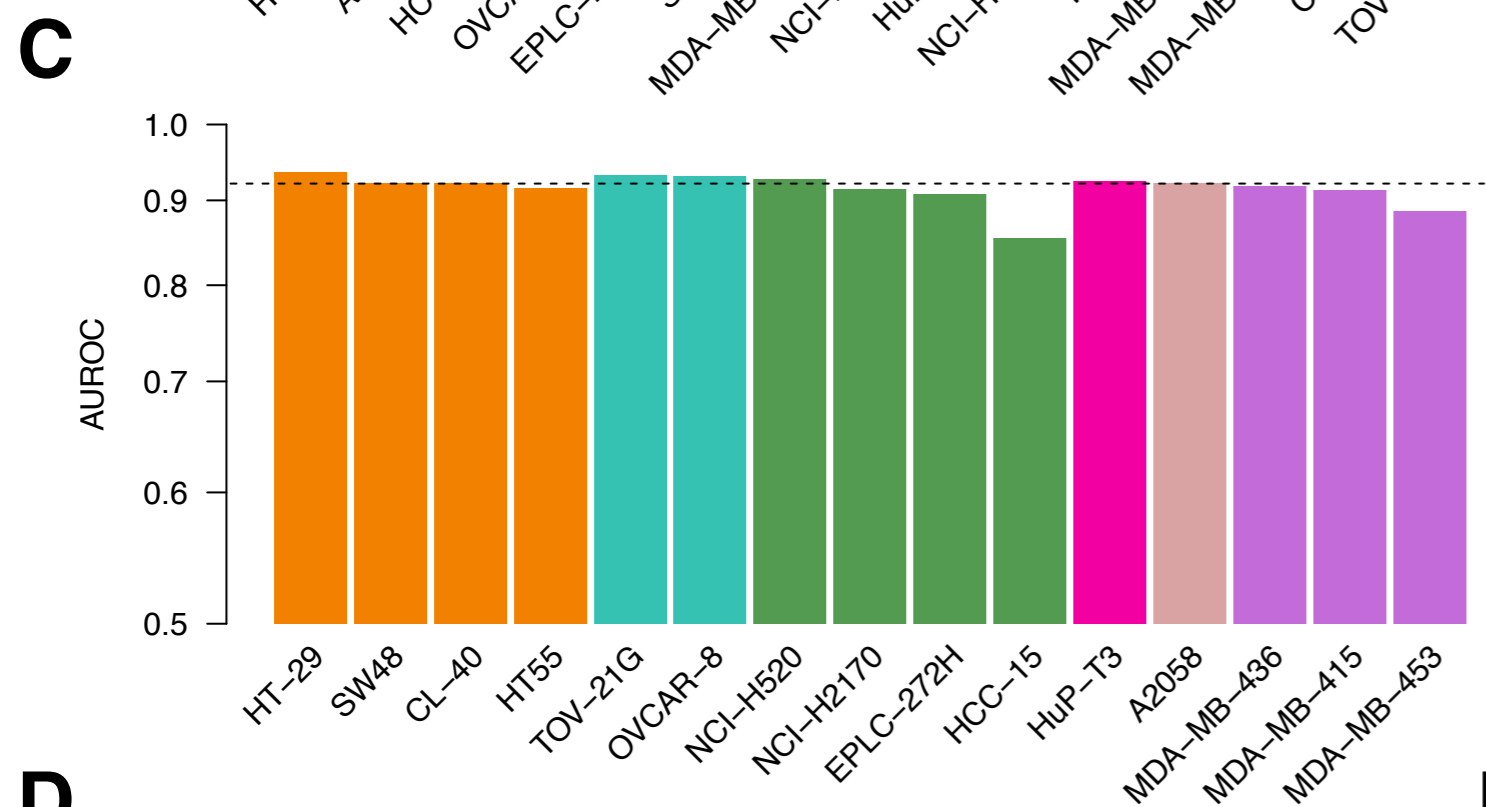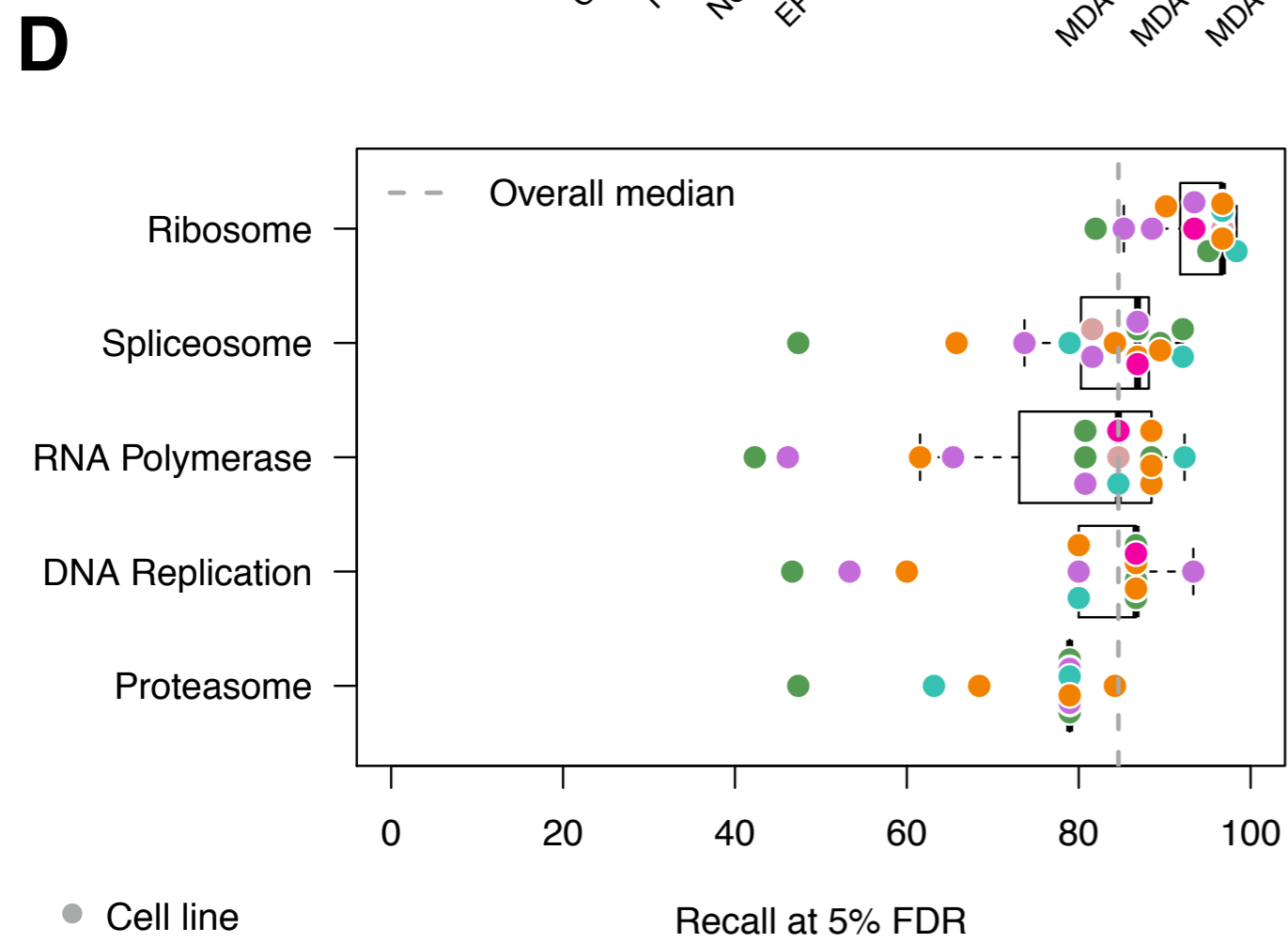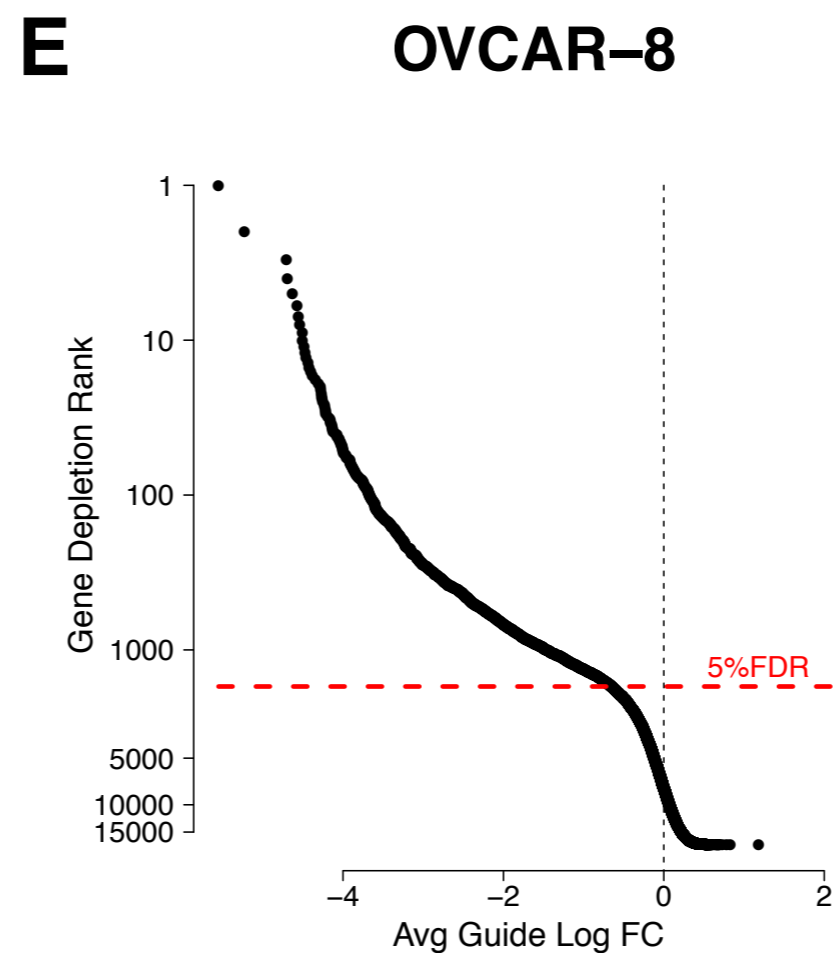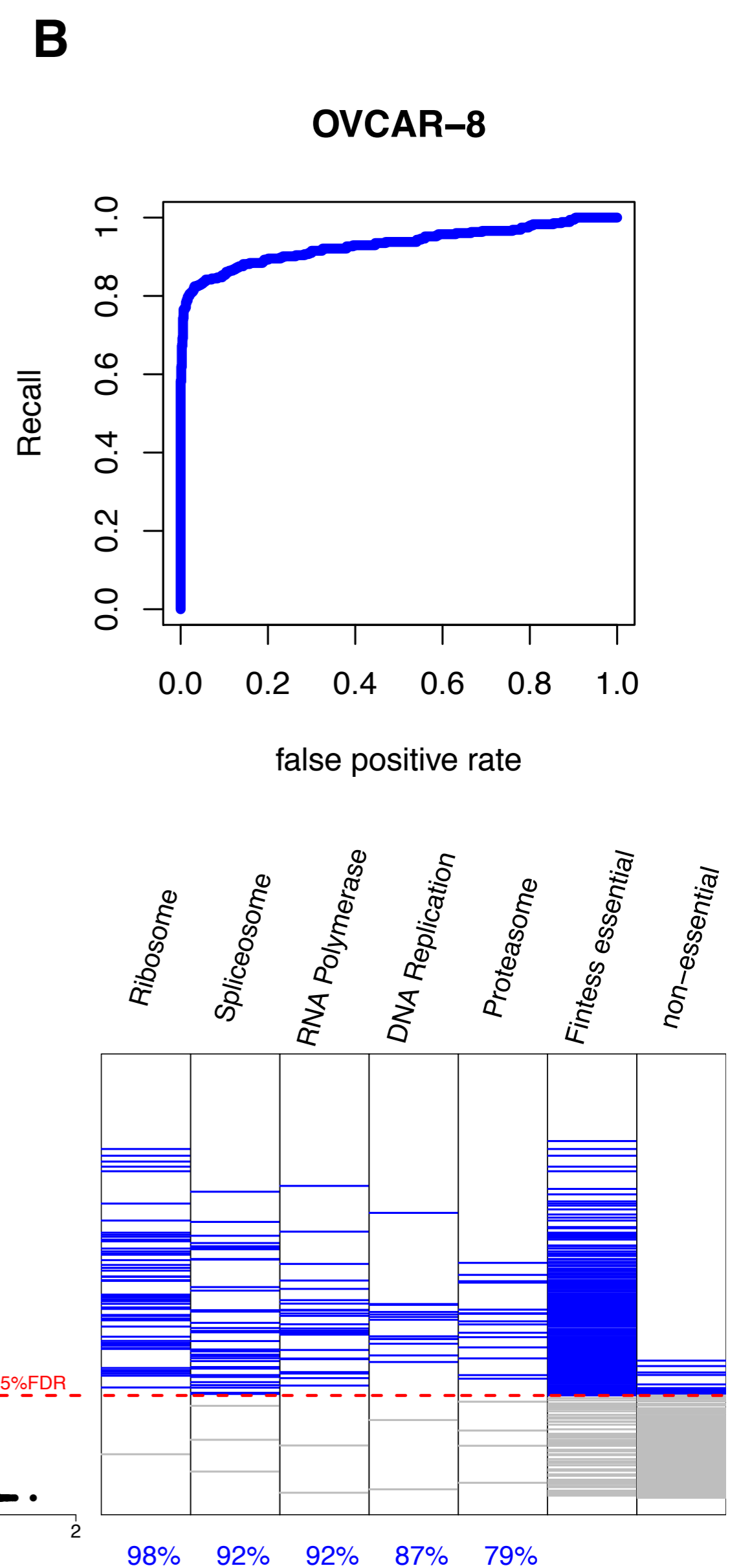

Supplement: Supplementary file 1 — Table S1. Project Score cell lines included in the study with annotations and screening description. Table S2. Quantification of copy number-associated bias before and after CRISPRcleanR correction. Table S3. Recall reduction following CRISPRcleanR correction across control gene-sets and cell lines. Table S4. Recall reduction post CRISPRcleanR correction across controls (mean-variance modeling). Table S5. Cancer driver gene dependencies following CRISPRcleanR correction. Table S6. List of gene signatures downloaded from MSigDB and used as positive controls. (ZIP 6330 kb) [file 12864_2018_4989_MOESM1_ESM.zip › Supp.Figure S1.pdf]

## EPLC-272H

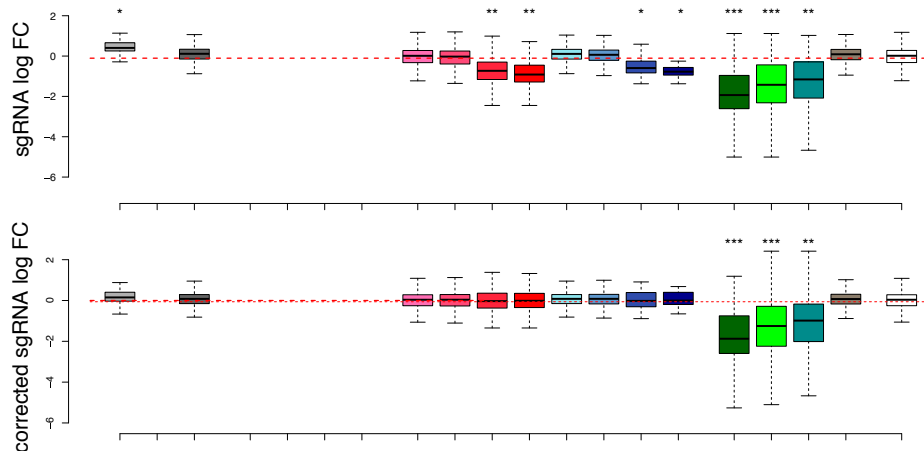

## HT-29

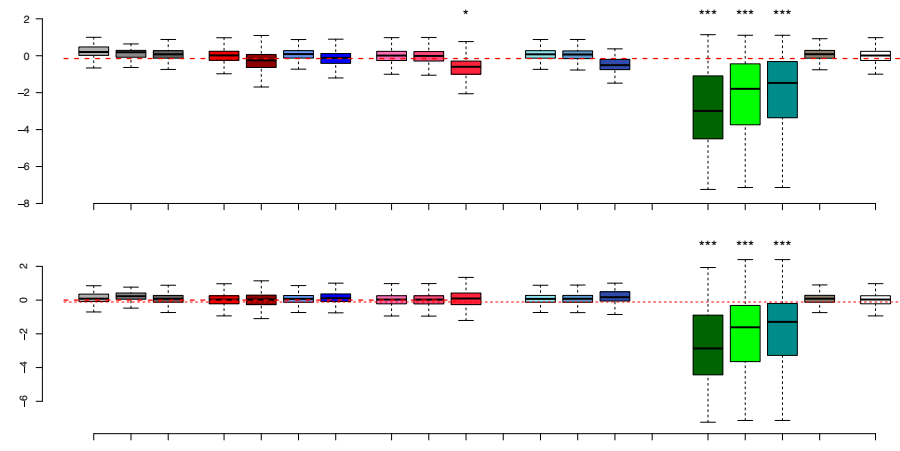

## HT55

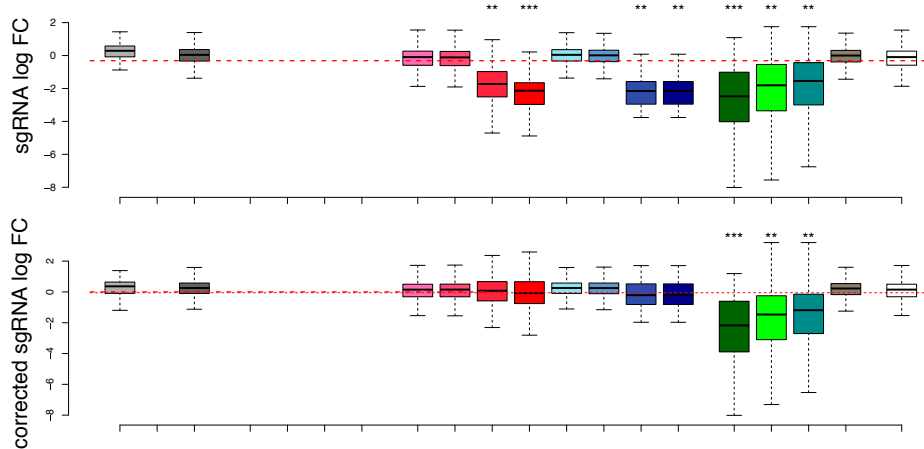

## HuP-T3

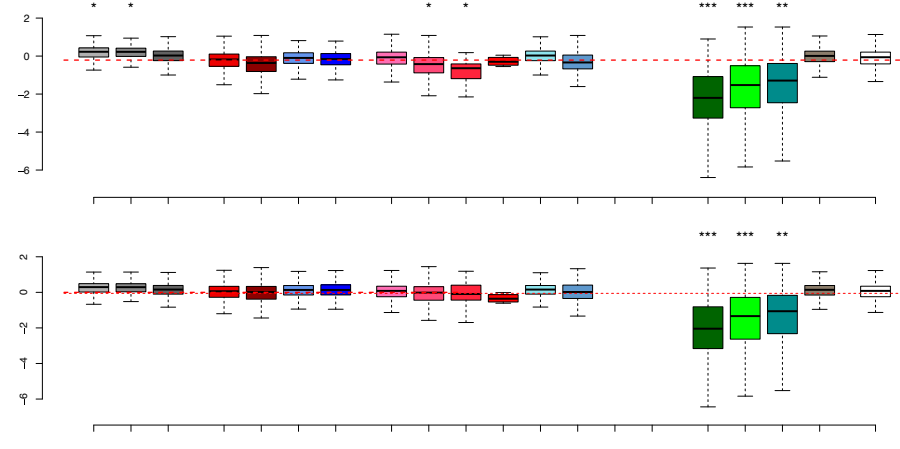

## MDA-MB-415

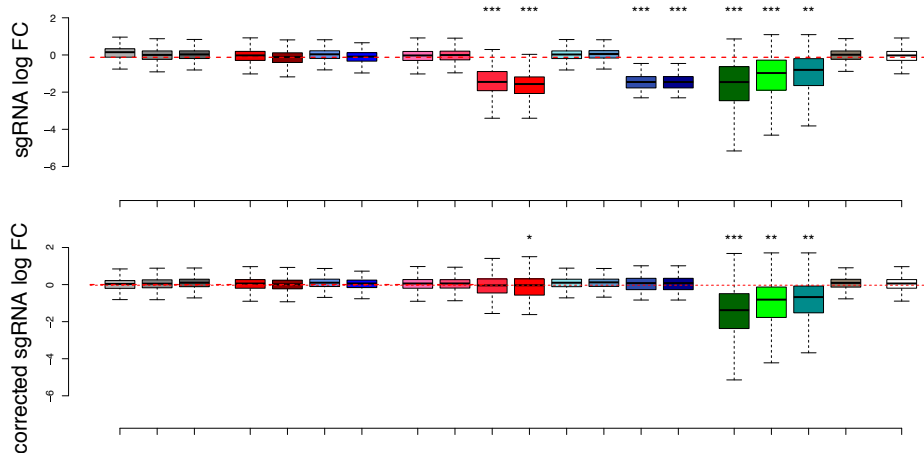

## MDA-MB-453

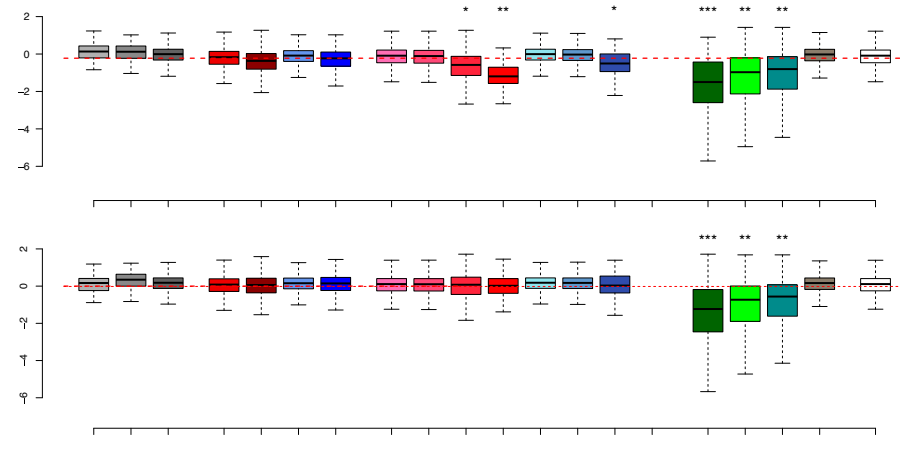

## NCI-H520

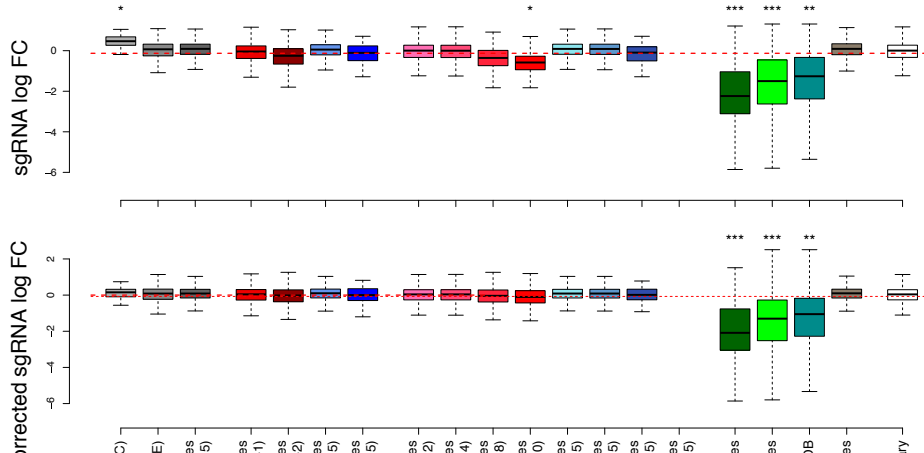

## NCI-H2170

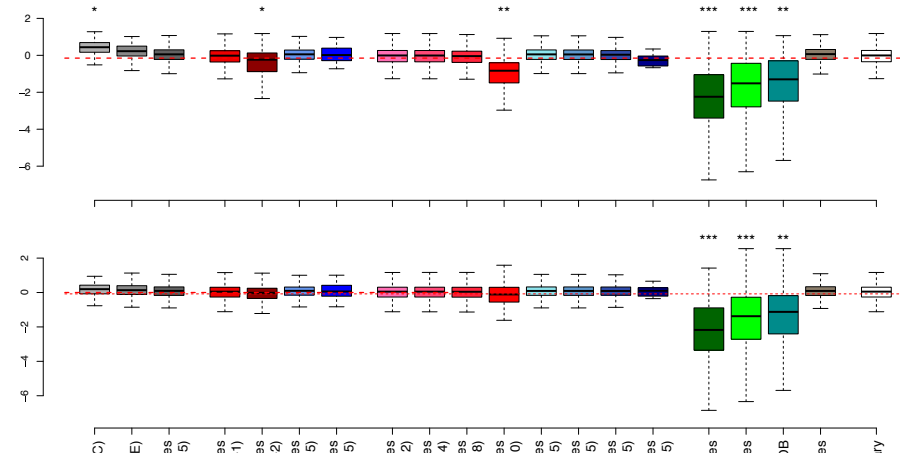

\* p < 0.05, Cohen's D > 0.5

\*\* p < 0.05, Cohen's D > 1

\*\*\* p < 0.05, Cohen's D > 2

Supplement: Supplementary file 1 — Table S1. Project Score cell lines included in the study with annotations and screening description. Table S2. Quantification of copy number-associated bias before and after CRISPRcleanR correction. Table S3. Recall reduction following CRISPRcleanR correction across control gene-sets and cell lines. Table S4. Recall reduction post CRISPRcleanR correction across controls (mean-variance modeling). Table S5. Cancer driver gene dependencies following CRISPRcleanR correction. Table S6. List of gene signatures downloaded from MSigDB and used as positive controls. (ZIP 6330 kb) [file 12864_2018_4989_MOESM1_ESM.zip › Supp.Figure S2.pdf]

**EPLC-272H****HT55****MDA-MB-415**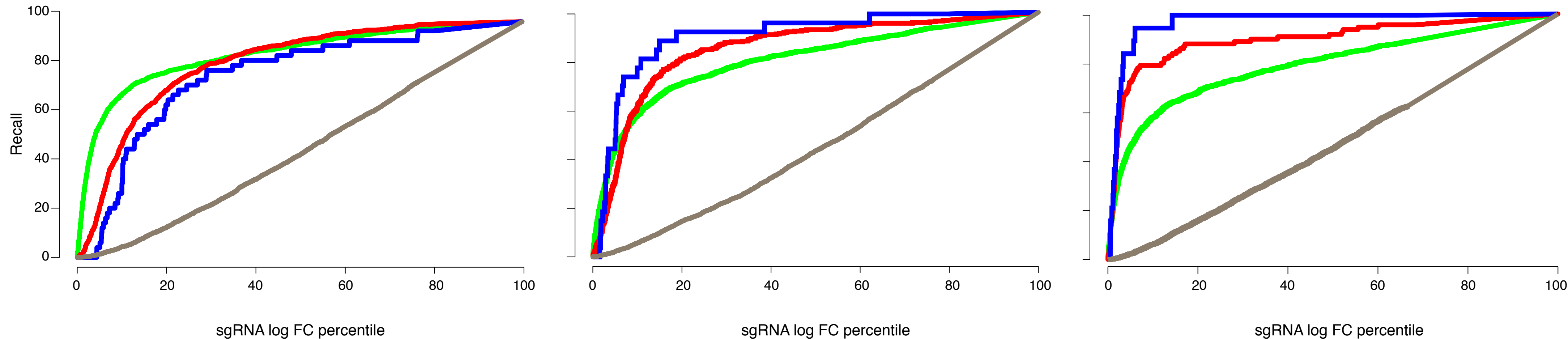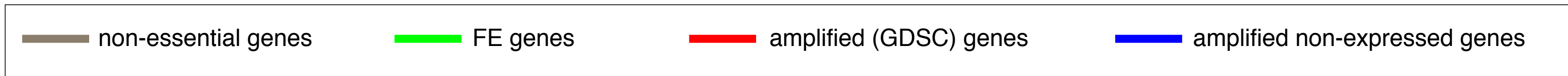

Supplement: Supplementary file 1 — Table S1. Project Score cell lines included in the study with annotations and screening description. Table S2. Quantification of copy number-associated bias before and after CRISPRcleanR correction. Table S3. Recall reduction following CRISPRcleanR correction across control gene-sets and cell lines. Table S4. Recall reduction post CRISPRcleanR correction across controls (mean-variance modeling). Table S5. Cancer driver gene dependencies following CRISPRcleanR correction. Table S6. List of gene signatures downloaded from MSigDB and used as positive controls. (ZIP 6330 kb) [file 12864_2018_4989_MOESM1_ESM.zip › Supp.Figure S3.pdf]

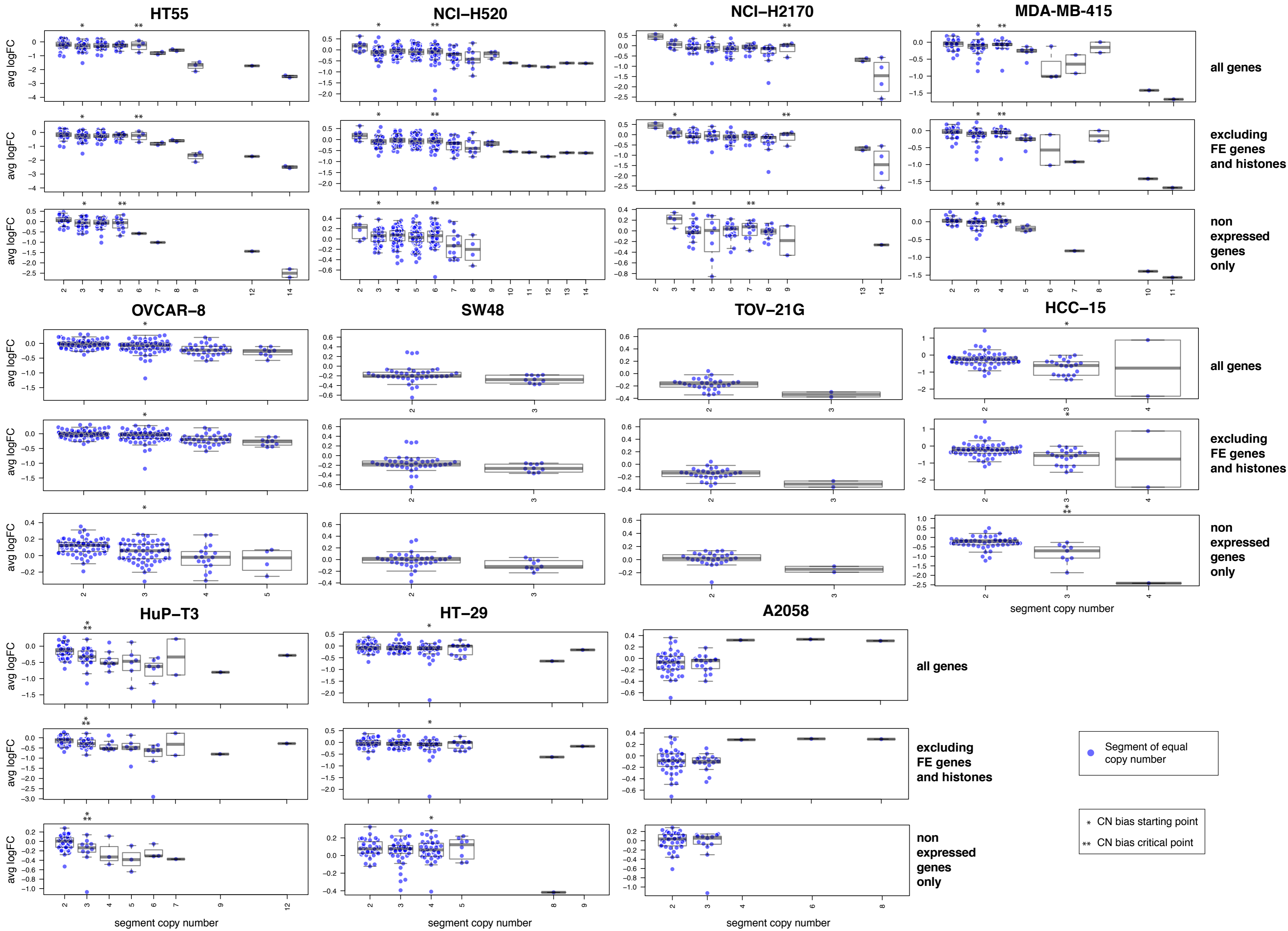

Supplement: Supplementary file 1 — Table S1. Project Score cell lines included in the study with annotations and screening description. Table S2. Quantification of copy number-associated bias before and after CRISPRcleanR correction. Table S3. Recall reduction following CRISPRcleanR correction across control gene-sets and cell lines. Table S4. Recall reduction post CRISPRcleanR correction across controls (mean-variance modeling). Table S5. Cancer driver gene dependencies following CRISPRcleanR correction. Table S6. List of gene signatures downloaded from MSigDB and used as positive controls. (ZIP 6330 kb) [file 12864_2018_4989_MOESM1_ESM.zip › Supp.Figure S4.pdf]

# Recall at 5% FDR across 342 cell lines (Project Achilles Data - Meyers 2017)

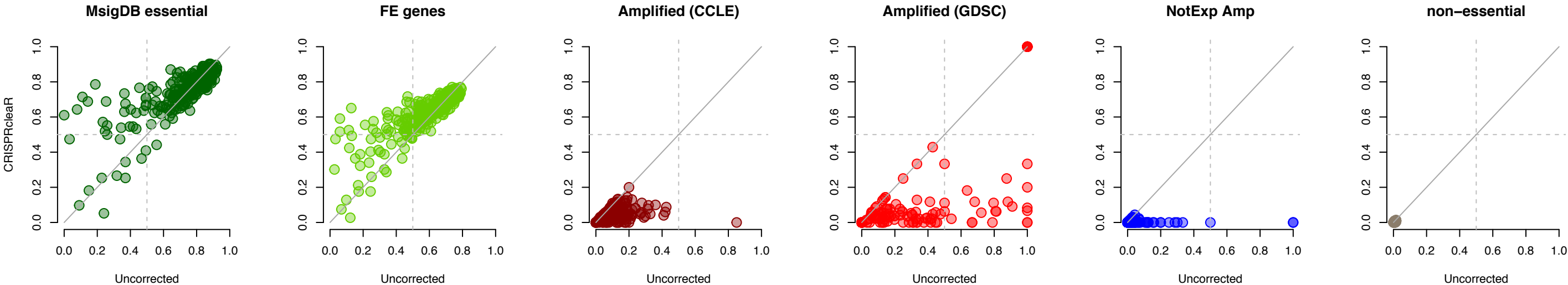

Supplement: Supplementary file 1 — Table S1. Project Score cell lines included in the study with annotations and screening description. Table S2. Quantification of copy number-associated bias before and after CRISPRcleanR correction. Table S3. Recall reduction following CRISPRcleanR correction across control gene-sets and cell lines. Table S4. Recall reduction post CRISPRcleanR correction across controls (mean-variance modeling). Table S5. Cancer driver gene dependencies following CRISPRcleanR correction. Table S6. List of gene signatures downloaded from MSigDB and used as positive controls. (ZIP 6330 kb) [file 12864_2018_4989_MOESM1_ESM.zip › Supp.Figure S6.pdf]

A

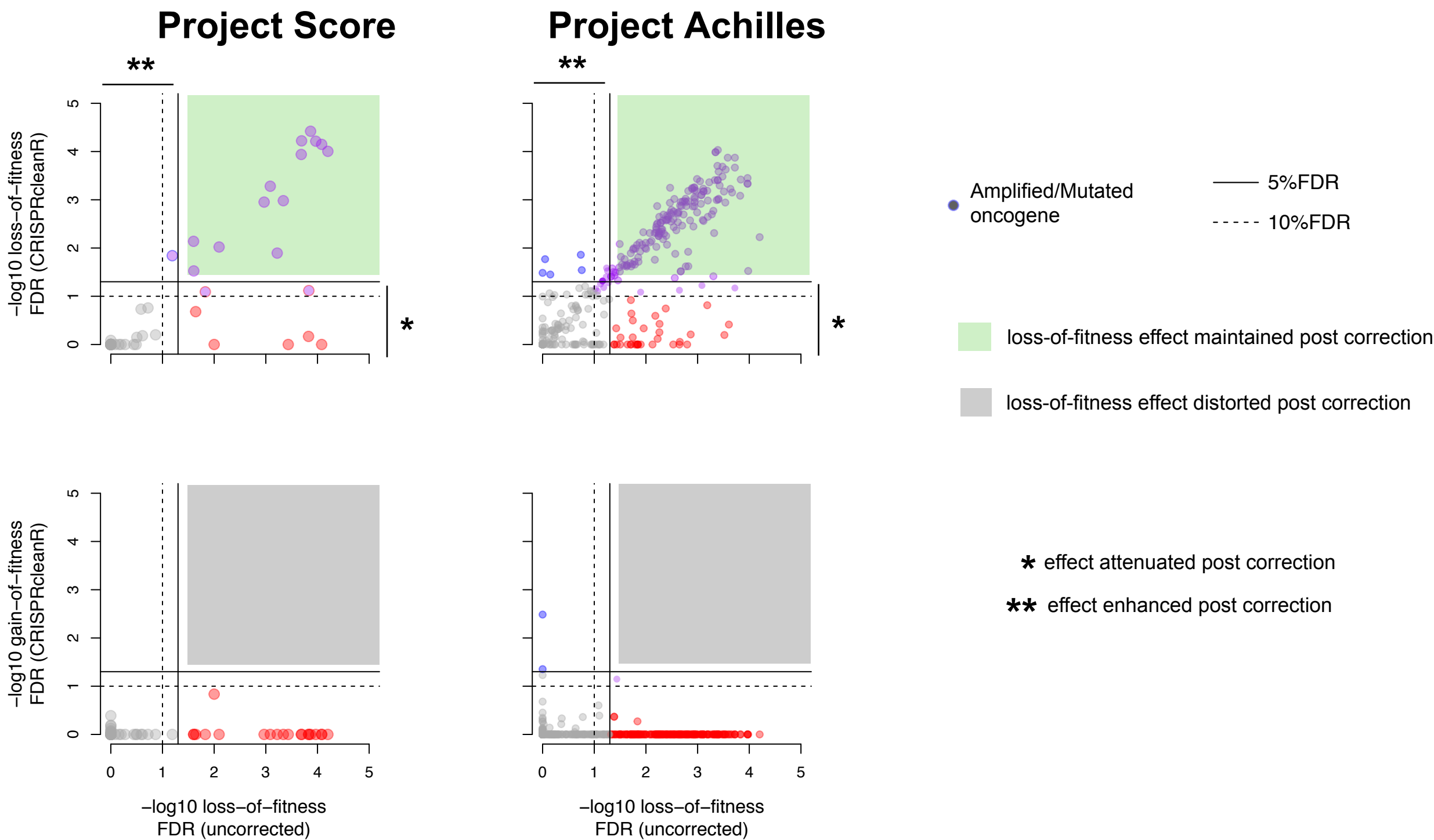

B

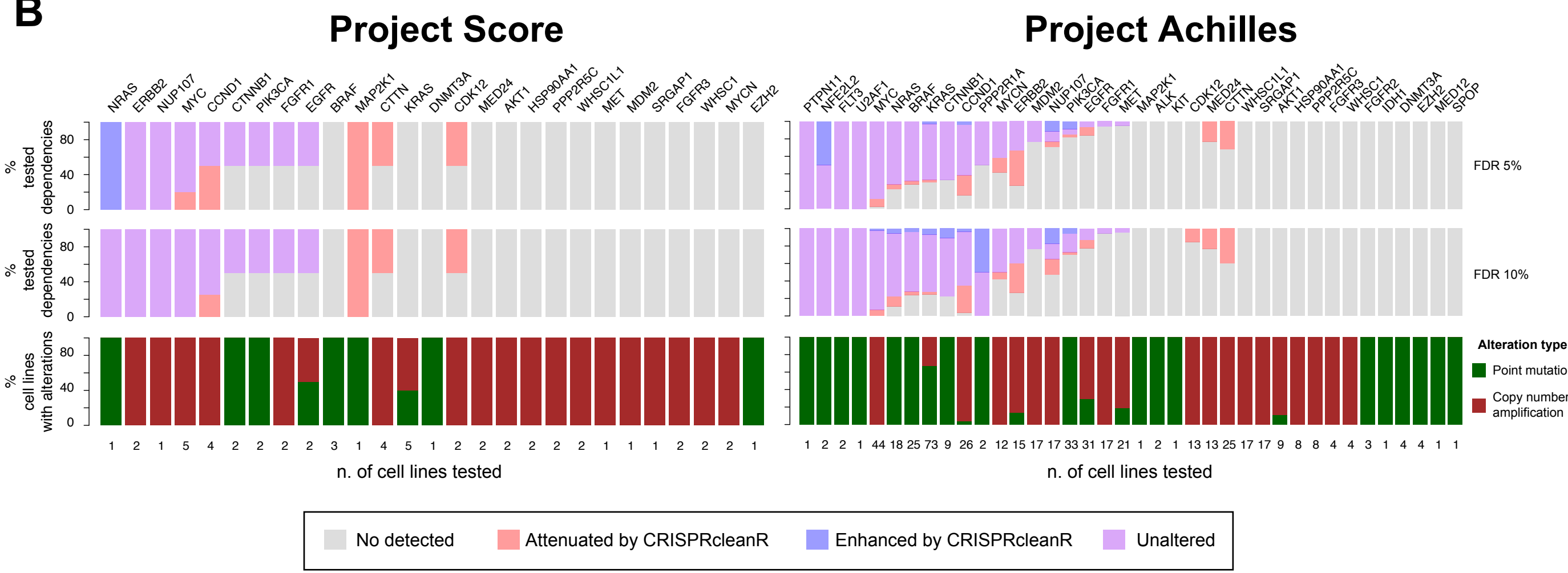

Supplement: Supplementary file 1 — Table S1. Project Score cell lines included in the study with annotations and screening description. Table S2. Quantification of copy number-associated bias before and after CRISPRcleanR correction. Table S3. Recall reduction following CRISPRcleanR correction across control gene-sets and cell lines. Table S4. Recall reduction post CRISPRcleanR correction across controls (mean-variance modeling). Table S5. Cancer driver gene dependencies following CRISPRcleanR correction. Table S6. List of gene signatures downloaded from MSigDB and used as positive controls. (ZIP 6330 kb) [file 12864_2018_4989_MOESM1_ESM.zip › Supp.Figure S9.pdf]
